# Cognitive behaviour therapy for fatigued cancer survivors: long-term follow-up

**DOI:** 10.1038/sj.bjc.6603899

**Published:** 2007-07-24

**Authors:** M F M Gielissen, C A H H V M Verhagen, G Bleijenberg

**Affiliations:** 1 Expert Centre Chronic Fatigue Nijmegen, Radboud University Nijmegen Medical Centre, Nijmegen 6500 HB, The Netherlands; 2 Department of Medical Oncology, Radboud University Nijmegen Medical Centre, Nijmegen 6500 HB, The Netherlands

**Keywords:** cancer survivors, cognitive behaviour therapy, fatigue, intervention, long-term effect, post-cancer fatigue

## Abstract

An earlier randomised-controlled trial demonstrated the positive effects of cognitive behaviour therapy (CBT), especially designed for fatigued cancer survivors in reducing fatigue, functional impairments and psychological distress. In the current prospective study, we were able to examine the long-term effect of CBT in patients who completed the therapy. Predictors of fatigue severity at follow-up were exploratory investigated. Sixty-eight patients who completed CBT were assessed at pretreatment, post-treatment and at follow-up (mean follow-up 1.9 years (s.d. 1.0), range: 1–4 years). To analyse possible predictors of treatment outcome a linear regression (enter) was carried out. Improvements on fatigue severity, functional impairment and psychological distress after CBT appeared to remain stable during a follow-up period. Patients who were not fatigued anymore at follow-up were not different from a reference group of non-fatigued cancer survivors. The explorative regression analysis showed that fatigue severity, psychological distress and somatic attributions at pretreatment contributed to persistent fatigue severity at follow-up. Cognitive behaviour therapy, especially designed for post-cancer fatigue, is successful in reducing fatigue and functional impairment in cancer survivors. Moreover, these positive effects were maintained at about 2 years after finishing CBT.

Fatigue is a common and distressing side effect of cancer treatment (Servaes *et al*, 2002b; Prue *et al*, 2006). Unfortunately, fatigue persists in patients for even years after completion of curative treatment. At least a quarter of the cancer survivors suffer from post-cancer fatigue, with profound effects on quality of life (Servaes *et al*, 2002b, in press; Hjermstad *et al*, 2005; Sugawara *et al*, 2005; Bower *et al*, 2006; Prue *et al*, 2006).

Although research on post-cancer fatigue has increased in the last decennia, there are only a few randomised-controlled trials (RCTs) investigating the management of post-cancer fatigue. Until now, six RCTs have investigated the effect of an intervention on fatigue, measured as a primary or secondary outcome. Two were pilot studies and found no effect on fatigue (Basen-Engquist *et al*, 2006; Culos-Reed *et al*, 2006). No effect was found investigating a lifestyle physical activity intervention (Basen-Engquist *et al*, 2006) and the second study found no effect of yoga (Culos-Reed *et al*, 2006). Two studies investigated the effect of exercise in cancer survivors. Both studies used fatigue as a secondary outcome and showed beneficial effects (Courneya *et al*, 2003; Pinto *et al*, 2005). The fifth study found that acupuncture was a more effective method to improve fatigue compared with acupressure or sham acupressure (Molassiotis *et al*, in press). None of these RCTs includes follow-up assessments. In the last RCT cognitive behaviour therapy (CBT), especially designed for post-cancer fatigue, appeared to be highly effective (Gielissen *et al*, 2006). The rationale of this intervention was based on the model of precipitating and perpetuating factors. Fatigue seems to be elicited during the treatment phase, but later on there is no clear relationship between persistent fatigue and initial disease and cancer treatment variables (Servaes *et al*, 2002b; Hjermstad *et al*, 2005; Ng *et al*, 2005; Prue *et al*, 2006; Young and White, 2006). The assumption is that the cancer itself and/or the cancer treatment may have triggered fatigue (precipitating factors), but other factors are responsible for persistence of fatigue complaints (perpetuating factors). Cognitive behaviour therapy for post-cancer fatigue is focused on these perpetuating factors. The RCT consisted of two conditions, the intervention condition (6 months of CBT) and waiting list condition (6 months). Patients in the intervention condition reported a clinically relevant decrease compared to patients in the waiting list condition in fatigue severity, functional impairment and psychological distress. Patients in the waiting list condition were informed beforehand that, if desired, they could start therapy directly after the waiting period of 6 months.

In this current study, the long-term effect of CBT will be investigated in patients who were involved in this former study and received CBT, including patients in the intervention condition and patients who had been treated after the 6-month waiting list. Furthermore, we will exploratory investigate predictors of fatigue severity at follow-up.

## METHODS

### Sample

Between December 2001 and September 2004, six departments of the Radboud University Nijmegen Medical Centre participated in the recruitment of patients for this study. Cancer survivors who experienced severe fatigue (score of 35 or higher on the Checklist Individual Strength, fatigue subscale) were recruited from the outpatient clinics of medical oncology, urology, surgery, orthopaedic, haematology and gynaecology. During follow-up visits in the hospital fatigued survivors were screened by their physician on clinically relevant systematic diseases (eg, malnutrition, haemoglobin level, presence of hypothyroidism and other physical comorbidities). If a physician was certain that the fatigue had no somatic cause, the patient was invited to participate. Patients completed curative treatment for cancer at least 1 year ago and had a minimal age at disease onset of 18 years. At time of participation patients had no evidence of disease recurrence and patients were not older than 65 years. Patients with current psychological or psychiatric treatment were excluded. The ethics committee of the hospital approved the study.

### Intervention

Cognitive behaviour therapy was focused on six perpetuating factors of post-cancer fatigue, which were based on existing literature and experience in clinical practice. They involve (1) insufficient coping with the experience of cancer, (2) fear of disease recurrence (Servaes *et al*, 2003; Young and White, 2006), (3) dysfunctional cognitions concerning fatigue (Broeckel *et al*, 1998; Servaes *et al*, 2002c), (4) dysregulation of sleep (Servaes *et al*, 2002b; Prue *et al*, 2006), (5) dysregulation of activity (Servaes *et al*, 2002a; Prue *et al*, 2006; Young and White, 2006) and (6) low social support and negative social interactions (Servaes *et al*, 2002c).

Each perpetuating factor became a module in the therapy protocol. Because of the existence of large differences within the group of fatigued cancer survivors (Servaes *et al*, 2002a), therapy was adapted to each individual. To determine which modules were necessary, each perpetuating factor was measured with specific questionnaires. If a patient scored problematic on one of these questionnaire, the accessory module became part of the treatment, resulting in an individualised treatment protocol per patient. It is important to realise that the therapy only varied in number of modules, but within each module the therapy is standardised. The number of sessions was determined by the number of used modules and by reaching the goal of the therapy.

Three therapists with previous CBT experience in patients with chronic fatigue treated patients who started directly with CBT as well as patients who started CBT after the waiting list period. For a more detailed description of the intervention see Gielissen *et al* (2006) (see Appendix A).

### Assessment

Patients were asked to complete questionnaires at the Expert Centre Chronic Fatigue of the Radboud University Nijmegen Medical Centre, pretreatment and post-treatment. Additionally, a package of questionnaires was sent by mail to all patients 6 months after the last patient finished CBT.

#### Outcome measures

*Fatigue severity* was measured by the fatigue severity subscale of the checklist individual strength (CIS) (Vercoulen *et al*, 1994; Beurskens *et al*, 2000; Dittner *et al*, 2004). The questionnaire has been used in cancer survivors (Servaes *et al*, 2002a, 2002c, 2003, in press; Gielissen *et al*, 2006), showed good reliability, discriminative validity and sensitivity to change (Beurskens *et al*, 2000; Prins *et al*, 2001; Stulemeijer *et al*, 2005; Gielissen *et al*, 2006).

*Functional impairment* was measured by the sickness impact profile-8 (SIP-8). This widely used measure has good reliability and content validity (Bergner *et al*, 1981; Jacobs *et al*, 1990).

*Psychological distress* was measured by the symptom check list 90, which has good reliability and discriminating validity (Arindell and Ettema, 1986; Derogatis, 1994).

#### Perpetuating factors

*Coping with the experience of cancer* was measured with the Dutch version of the Impact of Event Scale, which measures the extent to which a subject is currently occupied with the coping process after a major event (in this study the diagnose and treatment for cancer) (Brom and Kleber, 1985; Creamer *et al*, 2003; van der Ploeg *et al*, 2004).

*Fear of disease recurrence* was measured by two items of the Cancer Acceptance Scale (CAS) (Servaes *et al*, 2003).

*Cognitions related to fatigue.* Self-efficacy was measured with the Self-Efficacy Scale (Vercoulen *et al*, 1998; Prins *et al*, 2001; Servaes *et al*, 2002c, 2003) and somatic related attributions with regard to fatigue complaints were measured with the Causal Attribution List (CAL) (Servaes *et al*, 2002c).

*Sleep disturbances* was measured with the sleep/rest subscale of the SIP-8, and the insomnia subscale of the Quality of Life Questionnaire-C30 (QLQ-C30) (Aaronson *et al*, 1993).

*Physical activity* was measured with the physical functioning and role functioning subscale of the QLQ-C30. Furthermore, physical activity was measured with the subscales home management, work and recreation and pastimes from the SIP.

*Social functioning* was measured with the van Sonderen Social Support Inventory (SSL) (van Sonderen, 1993).

### Statistical analysis

Data analyses were performed using SPSS (version 12.1). Independent samples *t*-test and *χ*^2^-squared tests were performed testing differences between the intervention condition and the waiting list condition.

In the current study, the data collected at the end of the 6-month waiting period were used as pretreatment measurements. Comparison of the results of the pretreatment, post-treatment and follow-up assessments were carried out by GLM repeated measures analysis. Furthermore, GLM multivariate analysis was performed testing the differences between different follow-up periods and with a reference group. In a previous study of our research group 93 non-fatigued breast cancer patients were identified and used in this study as reference group (CIS<35; mean age, 46.4 years; s.d.=6.3; Servaes *et al*, 2002c).

Mann–Whitney *U*-tests were used testing the differences between patient who did not accept CBT after the 6-month waiting list period and patients who completed CBT.

McNemar tests were used to analyse the differences between the proportions of patients who did not meet the criteria for severe fatigue (CIS-fatigue<35) anymore at post-treatment and follow-up.

To analyse possible predictors of treatment effects a linear regression (enter) was carried out, with fatigue severity at the last follow-up assessment as dependent variable. Pearson correlations between fatigue severity at follow-up and the six perpetuating factors were used as preparatory analyses to examine the contribution of these factors to fatigue severity. Those measures that correlated significant with the fatigue severity at follow-up were used as independent variables in the logistic regression analyses. Correlations between the six perpetuating factors were tested on multicollinearity (*r*<0.9).

## RESULTS

### Sample

**Figure 1:**
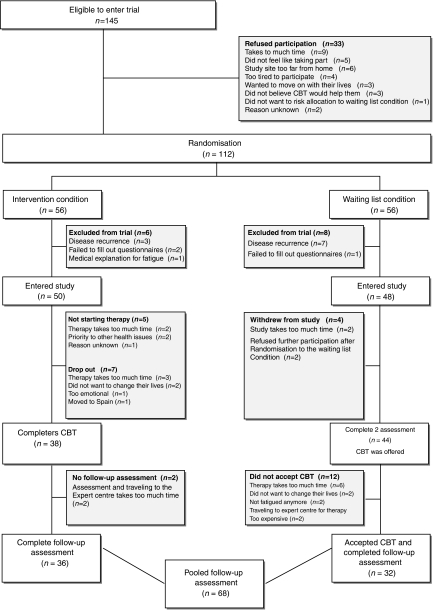
Trial profile.

Figure 1 shows the trial profile. The controlled data are described in Gielissen *et al* (2006). In this current study, we used the pooled data of both conditions. In the intervention condition, 38 patients completed CBT of whom 36 had a follow-up assessment. Forty-four patients completed the 6-month waiting list period and were offered CBT. Thirty-two patients accepted and completed the therapy and the follow-up assessment. There were no significant differences between patients in the intervention condition and waiting list condition on demographic and medical characteristics (Table 1). In addition, no significant differences were found on the outcome variables at pretreatment (fatigue severity *P*=0.052; functional impairment *P*=0.210; psychological distress *P*=0.300) and post-treatment (fatigue severity *P*=0.582; functional impairment 0.118; psychological distress *P*=0.346). Furthermore, the number of CBT sessions in both conditions were equal (12.5 (4.7) *vs* 12.4 (4.6), *P*=0.853). Because no differences were found, the data of both conditions were pooled (Table 1).

Furthermore, we compared patients who did not accept CBT after the 6-month waiting list period (*n*=12) with patients who completed CBT (*n*=68). There were no differences in the pretreatment assessment on fatigue severity (*P*=0.205), functional impairment (*P*=0.925) and psychological distress (*P*=0.671). Seven of the 12 patients who did not accept CBT after the waiting list period, completed the follow-up assessment.

### Long-term effect

The mean length of time between completion of therapy and follow-up assessment was 1.9 years (s.d.=1.0) with a range of 6 months to 4 years. The median was 2.0 years. The time interval between completion of therapy and follow-up assessment varied because patients entered the study at various times and started treatment at different moments.

Information about the outcome variables at the three assessments are listed in Table 2. Scores of fatigue severity, functional impairment and psychological distress significantly decreased at post-treatment and follow-up assessment compared with the pretreatment assessment. Additionally, the means on all outcome measures remained stable between post-treatment and follow-up.

Compared with the reference group, patients in this study were significantly more fatigued at follow-up assessment, but had the same level of functional impairment and psychological distress. The follow-up period of patients who did not accept CBT (*n*=7) was comparable with the follow-up period of patients who completed CBT (1.5 years (s.d.=0.8), *P*=0.145). Patients who did not accept CBT were significantly more fatigued, had more functional impairments and higher psychological distress at follow-up compared to patients who accepted CBT (Table 2).

Eighty-one percent (*n*=55) of the patients did not meet the criteria of severe fatigue at post-treatment (CIS-fatigue⩾35). At follow-up this percentage of non-fatigued patients was 71% (*n*=48, *P*=0.118). Compared with the non-fatigued reference group (Table 2), the patients who were not fatigued after CBT (*n*=48) had the same level of fatigue (19.9; s.d.=8.4, *P*=0.842), the same level of functional impairment (271.0; s.d.=292.7, *P*=0.476) and a significantly lower level of psychological distress (106.3; s.d.=14.4, *P*=0.042).

### Short- *vs* long-term follow-up

As there is a considerable range in the duration of the follow-up, we investigated whether the treatment outcome differed between patients with a shorter and a longer follow-up period. Patients were divided into four groups: patients who completed CBT between 6 months and 1 year ago (*n*=15), between 1 and 2 years ago (*n*=21), between 2 and 3 years ago (*n*=20), and between 3 and 4 years ago (*n*=12). *Post hoc* analyses showed no significant differences on change scores (pretreatment scores −/− follow-up scores) between the four-follow-up period on fatigue severity, functional impairment and psychological distress (Table 3). Furthermore, correlations between time since CBT and fatigue severity (*r*=−0.067, *P*=0.585), functional impairment (*r*=216, *P*=0.077) and psychological distress (*r*=141, *P*=0.251) were low and nonsignificant.

### Predictors

Results of the preparatory analyses indicated that fatigue at follow-up was significantly correlated with fatigue severity (CIS-fatigue, *r*=0.354, *P*=0.003), psychological distress (SCL90-total, *r*=0.398, *P*=0.001), somatic related attributions (CAL, *r*=0.293, *P*=0.015) and insufficiency in social interactions (SSL-D, *r*=0.316, *P*=0.009) at pretreatment. These variables were used as independent variables in the linear regression analysis. Table 4 summarise the regression analysis. Somatic attributions contributed almost significantly (*P*=0.050) to fatigue severity at follow-up. Furthermore, a trend was seen for pretreatment fatigue severity (*P*=0.064) and psychological distress (*P*=0.074).

## DISCUSSION

The results of the present study indicate that the positive results of CBT especially designed for fatigued cancer survivors were maintained at follow-up. Fatigue severity, functional impairment and psychological distress remained stable in patients who completed CBT after almost a mean follow-up period of 2 years. Furthermore, we could not find any difference between patients with a short- and a long-term follow-up. Therefore, even after 4 years the positive effect of CBT remained.

Patients who were allocated to the 6-month waiting list were offered CBT directly after the second assessment. Therefore, the long-term effect was investigated with an uncontrolled design. Nevertheless, patients who were recovered at follow-up were comparable with a reference group of non-fatigued cancer survivors. Additionally, we investigated a small group of patients who did not accept CBT after the waiting list period. These patients did not improve over time on fatigue severity, functional impairment and psychological distress. Because of the small sample size, we should be careful in interpreting these results. Patients could have improved regardless of the followed treatment. It would increase the impact of our findings if future studies could prove the long-term superiority of CBT over natural course in fatigued cancer survivors. Another reason why it is difficult to draw firm conclusions is that follow-up data were not available of all patients who participated in the previous RCT.

The explorative regression analysis showed a trend that patients with more fatigue, higher psychological distress and stronger somatic attributions at pretreatment were more fatigued at the follow-up assessment. Fatigued cancer survivors have the tendency to attribute their fatigue complaints to the cancer itself and/or cancer treatment (Servaes *et al*, 2002c). However, research on post-cancer fatigue fails to show such relationship, which makes this a false attribution (Servaes *et al*, 2002b; Hjermstad *et al*, 2005; Ng *et al*, 2005; Prue *et al*, 2006; Young and White, 2006). In the current model of post-cancer fatigue, we assume that fatigue originates in the diagnostic and treatment stage; however, there is no clear relationship between fatigue long after curative treatment and the initial disease and cancer treatment characteristics. Because somatic attributions still proved to contribute to fatigue at follow-up in this study, it seems that this aspect has received not enough attention during the CBT. If a patients continues to think that the cancer itself and/or cancer treatment is responsible for the experienced fatigue, the chance on recovery is lowered. It is possible that (further) education on post-cancer fatigue for professionals working in cancer care can increase the chance of improvement with CBT. Somatic attributions in fatigued cancer survivors can be reinforced by inaccurate information delivery about the cause of post-cancer fatigue. Therefore, education should be aimed particularly on the model of precipitating and perpetuating factors.

Furthermore, indications were found that patients with high psychological distress had a worse treatment outcome. Extreme high scores on the SCL90 total score (>200) are indicative for psychiatric comorbidity (Arindell and Ettema, 1986). Five patients in our sample met this criterion. All five patients remained fatigued after CBT. When deleting these cases, the trend of psychological distress as contributor to fatigue at follow-up disappeared in the regression analysis (*P*=0.776). Therefore, fatigued cancer survivors with high scores on psychological distress (probably indicative of psychiatric comorbidity) proved to have hardly any chance to improve with CBT for post-cancer fatigue. However, results from the regression analyses should be regarded as exploratory and interpreted with caution.

Most studies on post-cancer fatigue do not find an association between fatigue and cancer type (Servaes *et al*, 2002b; Prue *et al*, 2006; Gielissen *et al*, 2007). In the current study, we did not find a significant difference in fatigue severity at baseline between the different types of cancer (*P*=0.821). There was also no difference in effect of CBT on fatigue severity (*P*=0.983). However, our study was limited to patients with rather frequently diagnosed tumours. Therefore, replication is necessary in survivors with other cancer types.

The long-term follow-up results of our study shows that the positive effects of CBT especially designed for post-cancer fatigue are maintained even years after treatment. Until now, no other interventions have been published with comparable good results on post-cancer fatigue on the long term.

## Figures and Tables

**Table 1:** Characteristics of study participants

	**CBT (*n*=36)**	**Waiting list (*n*=32)**	**Pooled group (*n*=68)**
Age (years)	43.8 (10.3)	43.9 (10.3)	43.8 (10.2)
M/F (*n*)	19/17	16/16	35/33
			
*Cancer diagnosis* % (n)			
Mamma carcinoma	36% (13)	25% (8)	31% (21)
Testicular cancer	33% (12)	25% (8)	29% (20)
Haematological cancer	17% (6)	16% (5)	16% (11)
Other solid tumours	14% (5)	34% (11)	24% (16)
			
*Treatment type* % (n)^a^			
Surgery	75% (27)	81% (26)	78% (53)
Chemotherapy	70% (24)	84% (27)	75% (51)
Radiotherapy	53% (19)	44% (14)	49% (33)
Duration of cancer treatment (months)	6.6 (7.1)	7.3 (6.3)	6.9 (6.7)
Time since cancer treatment (years)	5.2 (4.0)	5.1 (3.6)	5.1 (3.8)

CBT=cognitive behaviour therapy.

Values are means (s.d.) unless stated otherwise.

a Percentages do not add up to 100% because more options are possible.

**Table 2:** Means and s.d. of CBT completers at pretreatment, post-treatment and follow-up, a reference group of non-fatigued cancer survivors and non-accepters of CBT at follow-up assessment

***N*=68**	**A pretreatment**	**B post-treatment**	**C follow-up**	**D reference values (*n*=98)**	**Non-accepters CBT at follow-up (*n*=7)**
Fatigue severity	45.3 (7.7)^a^, ^b^, ^c^	24.3 (10.9)^c^, ^d^	26.9 (13.1)^c^, ^d^	19.6 (8.4)^a^, ^b^, ^d^	40.3 (14.8)^e^
Functional impairment	937.1 (530.4)^a^, ^b^, ^c^	415.1 (438.6)^d^	429.8 (483.2)^d^	309.5 (333.4)^d^	842.9 (302.2)^e^
Psychological well-being	138.5 (35.6)^a^, ^b^, ^c^	113.6 (25.5)^d^	119.3 (37.1)^d^	113.2 (20.3)^d^	138.6 (39.8)

CBT=cognitive behaviour therapy.

a Significantly different from post-treatment assessment (*P*<0.05).

b Significantly different from the follow-up assessment (*P*<0.05).

c Significantly different from the reference group (*P*<0.05).

d Significantly different from pretreatment assessment (*P*<0.05).

e Significantly different from follow-up assessment of CBT-completers.

**Table 3:** Change scores (pretreatment scores −/− follow-up scores) at different follow-up points

***N*=68**	**A 6 months – 1 year (*n*=15)**	**B 1–2 years (*n*=21)**	**C 2–3 years (*n*=20)**	**D 3–4 years (*n*=12)**
Fatigue severity	16.3 (13.0)	20.4 (13.1)	15.8 (12.2)	21.8 (11.9)
Functional impairment	507.1 (358.3)	557.2 (473.5)	473.5 (501.5)	476.3 (351.1)
Psychological well-being	26.0 (19.5)	18.9 (21.0)	11.3 (38.7)	24.3 (14.4)

There were no significant differences on change scores between the four-follow-up periods.

**Table 4:** Linear regression (enter) to predict fatigue severity at follow-up

	**Dependent variable CIS-fatigue at follow-up**	
**Independent variables (pretreatment measurements)**	***β***	***P*-value**
Fatigue (CIS-fatigue)	0.373	0.064
Psychological distress (SCL90-total)	0.087	0.074
Dysfunctional cognitions (somatic-CAL)	1.803	0.050
Social insufficiency (SSL-D)	0.086	0.422

CAL=Causal Attribution List; CIS=checklist individual strength; SCL=symptom checklist; SSI=social support inventory.

Adjusted *R^2^* 0.222.

**Table A1 tbla1:** **Table A1**Patients were asked to complete questionnaires at the Expert Centre Chronic Fatigue of the Radboud University Nijmegen Medical Centre, pretreatment and post-treatment.

**Questionnaires**		**Response format**	**Example questions**
Fatigue severity	Checklist Individual strength – subscale Fatigue Severity (8 items)	Seven-point Likert scale	I feel tired
		Range 8–56	I am rested
		A score of 35 indicates severe fatigue	Physically I feel exhausted
Functional impairment	SIP-8 Home management (10 items) Mobility (10 items) Alertness behaviour (10 items) Sleep/rest (7 items) Ambulation (12 items) Social interactions (20 items) Work (8 items) Recreation and pastimes (8 items)	Patients can mark a box behind each statement. A total score is calculated by addition the weights of items Range 0–5799	I am not doing any of the house cleaning that I would usually do (hm) I am not going out to visit people at all (si) I walk shorter distances or stop to rest often (amb) I react slowly to thing that are said (alert) I have difficulty doing activities involving concentration and thinking (alert)
Psychological distress	Symptom Check List 90 (90 items) Anxiety (10 items) Agoraphobia (7 items) Depression (16 items) Somatisation (12 items) Obsessive-compulsive behaviour (9 items) Interpersonal sensitivity (18 items) Hostility (6 items) Sleep (3 items)	5 point Likert scale Range 90–450	During the past 7 days about how much were you distressed or bothered by: Feeling fearful (anx) Feeling of worthlessness (depr) Numbness or tingling in parts of your body (som) Feeling that people are unfriendly or dislike you (int.sens) Nervousness or shakiness inside (anx)
Coping with the experience of cancer	Impact of Event Scale Intrusion (7 items) Avoidance (8 items)	6-point Likert Scale Range 13–52	I had dreams about it (intr) I tried not to think about it (avoid) I tried not to talk about it (avoid)
Fear of disease recurrence	Cancer Acceptance Scale	4-point Likert Scale Range 2–8	I am worried about a tumour relapse I am anxious about my health
Cognitions related to fatigue	Self Efficacy Scale (7 items)	4-point Likert Scale Range 7–28	Whatever I do, I cannot change my complaints I think I could positively influence my fatigue
	Causal Attribution List – subscale somatic attribution (4 items)	4-point Likert Scale Range 4–16	Do you think your complaints have to do with the anti-cancer treatment?
Sleep disturbance	SIP-8 – subscale Sleep/Rest	4-point Likert Scale	I sleep more during the day
	EORTC QLQ-C30 – subscale Insomnia (1 item)		Have you had trouble sleeping?
Physical activity	EORTC QLQ-C30 – subscale physical functioning (5 items) EORTC QLQ-C30 – subscale role functioning (2 items) SIP-8 – Home management SIP-8 – Work SIP-8 – Recreation and Pastimes	Yes/No; range 5–10 4-point Likert Scale; range 2–8 Patients can mark a box behind each statement.	Do you have trouble waling a long walk? Has your physical condition interfered with your family life? I am not doing any of the clothes washing At work, I make more mistakes than usually I am doing fewer community activities
Social functioning	Van Sonderen Social Support Inventory SSL-I: amount of social support (34 items) SSL-D: insufficiency of supporting interactions (34 items) SSL-N: amount of negative interactions (7 items)	4-point Likert Scale Range 34–136 Range 34–135 Range 7–28	Do you experience friendliness and sympathy in your contacts with other people? Do you talk problems over with other people?

## Appendix A

[Table tbla1]
